# Supplementary material for: A general role for TANGO1, encoded by MIA3, in secretory pathway organization and function
Source: J Cell Sci. 2021 Sep 7;134(17):jcs259075. doi: 10.1242/jcs.259075 (PMC8524724; doi:10.1242/jcs.259075)
Supplement: Supplementary information [file joces-134-259075-s1.pdf]

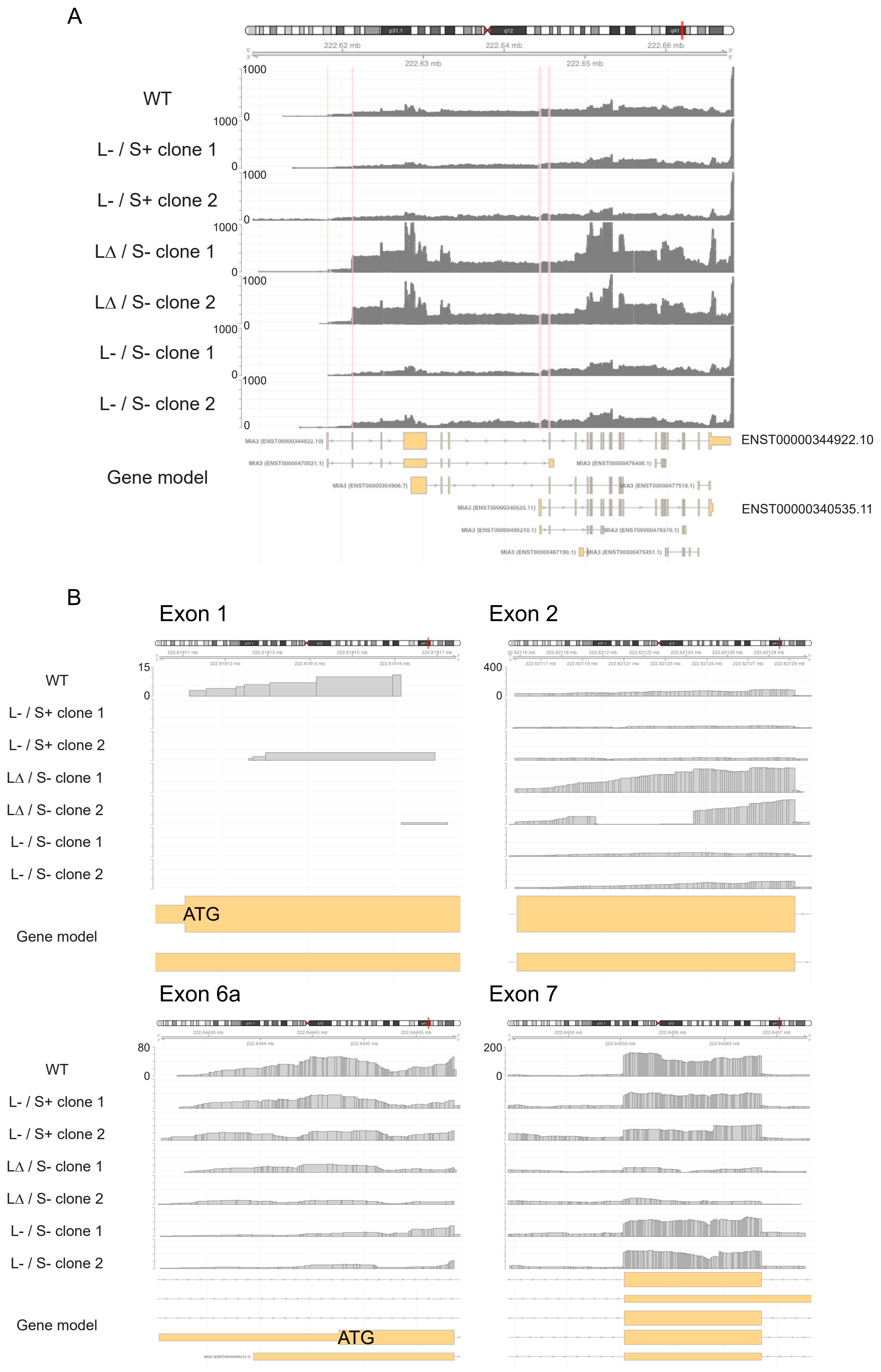

Fig. S1. RNAseq read mapping. A. Gene model for Mia3 gene (showing chr1:222,608,000 - 222,668,400) with read depth mapped for each cell line. B. Regions of particular interest (Exon 1, chr1:222,618,104 - 222,618,176; Exon 2, chr1:222,621,155 - 222,621,301; Exon 6a, chr1:222,644,301 - 222,644,593; Exon 7, chr1:222,645,442 - 222,645,734) are displayed is a chromosome ideogram, a genomic coordinate region track, and a genome annotation track showing putative gene models for MIA3. The gene annotation depicts predicted splice forms; ENST00000344922.10 denotes the major isoform TANGO1L, ENST00000340535.11 depicts TANGO1S. B. Exon 1 is significantly disrupted in all knockout cell lines with few if any reads around the core ATG site for TANGO1L. Exon 2 is also disrupted. Exon 6A shows the ATG start codon for TANGO1S. Exon 7 is the target of other gRNAs used. RNAseq data are derived from 3 independent RNA isolation and library preparations.

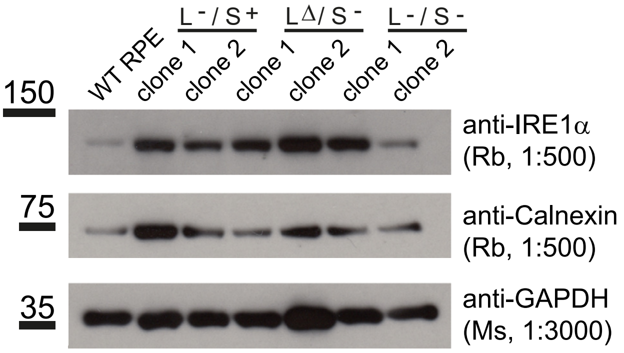

Fig. S2. Immunoblots show expression of IRE1a, calnexin, and GAPDH (as a loading control) in TANGO1 knockout cell lines. Uncropped blots at different exposures are included as Supplemental Data Set 8. Data are representative of n=3.

L- / S+

GO biological process complete	clone 1	clone 2	Average
regulation of transcription by RNA polymerase II (GO:0006357)	1.5	1.6	**1.6**
regulation of RNA metabolic process (GO:0051252)	1.37	1.45	**1.4**
regulation of macromolecule biosynthetic process (GO:0010556)	1.37	1.4	**1.4**
regulation of transcription, DNA-templated (GO:0006355)	1.36	1.45	**1.4**
regulation of nucleic acid-templated transcription (GO:1903506)	1.36	1.45	**1.4**
regulation of RNA biosynthetic process (GO:2001141)	1.36	1.45	**1.4**
regulation of nucleobase-containing compound metabolic process (GO:0019219)	1.36	1.4	**1.4**
regulation of cellular macromolecule biosynthetic process (GO:2000112)	1.36	1.41	**1.4**
regulation of biosynthetic process (GO:0009889)	1.35	1.37	**1.4**
regulation of cellular biosynthetic process (GO:0031326)	1.34	1.37	**1.4**
regulation of gene expression (GO:0010468)	1.25	1.28	**1.3**
regulation of nitrogen compound metabolic process (GO:0051171)	1.23	1.26	**1.2**
regulation of primary metabolic process (GO:0080090)	1.23	1.26	**1.2**
regulation of cellular metabolic process (GO:0031323)	1.22	1.24	**1.2**

LΔ / S-

GO biological process complete	clone 1	clone 2	Average
COPII-coated vesicle cargo loading (GO:0090110)	8.06	10.95	**9.5**
vesicle cargo loading (GO:0035459)	5.97	8.11	**7.0**
intra-Golgi vesicle-mediated transport (GO:0006891)	5.53	5.63	**5.6**
COPII vesicle coating (GO:0048208)	4.86	5.22	**5.0**
vesicle targeting, rough ER to cis-Golgi (GO:0048207)	4.86	5.22	**5.0**
COPII-coated vesicle budding (GO:0090114)	4.84	5.32	**5.1**
vesicle targeting, to, from or within Golgi (GO:0048199)	4.84	5.01	**4.9**
vesicle coating (GO:0006901)	4.81	4.91	**4.9**
IRE1-mediated unfolded protein response (GO:0036498)	4.4	5.18	**4.8**
retrograde vesicle-mediated transport, Golgi to endoplasmic reticulum (GO:000(	4.36	4.64	**4.5**
vesicle budding from membrane (GO:0006900)	4.07	3.83	**4.0**
vesicle targeting (GO:0006903)	4.03	3.81	**3.9**
endoplasmic reticulum to Golgi vesicle-mediated transport (GO:0006888)	3.62	4.14	**3.9**
Golgi organization (GO:0007030)	3.46	3.25	**3.4**
endoplasmic reticulum unfolded protein response (GO:0030968)	3.07	3.76	**3.4**

L- / S-

GO biological process complete	clone 1	clone 2
intra-Golgi vesicle-mediated transport (GO:0006891)	6.52	
retrograde vesicle-mediated transport, Golgi to endoplasmic reticulum (GO:000(	4.89	
zinc ion transport (GO:0006829)	4.79	
vesicle targeting, to, from or within Golgi (GO:0048199)	4.22	
COPII vesicle coating (GO:0048208)	4.05	
vesicle targeting, rough ER to cis-Golgi (GO:0048207)	4.05	
vesicle coating (GO:0006901)	4.01	
IRE1-mediated unfolded protein response (GO:0036498)	3.9	
COPII-coated vesicle budding (GO:0090114)	3.83	
vesicle targeting (GO:0006903)	3.65	
vesicle budding from membrane (GO:0006900)	3.52	
Golgi organization (GO:0007030)	3.48	
endoplasmic reticulum to Golgi vesicle-mediated transport (GO:0006888)	3.42	2.66
Golgi vesicle transport (GO:0048193)	3.12	
endoplasmic reticulum unfolded protein response (GO:0030968)	3.07	
post-Golgi vesicle-mediated transport (GO:0006892)	2.7	
establishment of vesicle localization (GO:0051650)	2.6	
cellular response to unfolded protein (GO:0034620)	2.58	
vesicle localization (GO:0051648)	2.54	
vesicle organization (GO:0016050)	2.16	2.03
glycoprotein biosynthetic process (GO:0009101)	2.06	
response to endoplasmic reticulum stress (GO:0034976)	2.03	

Fig. S3. Gene ontology analysis of pooled outcomes from MIA3 knockout cell lines. Tables show those terms enriched in each cell line. RNAseq data are derived from 3 independent RNA isolation and library preparations.

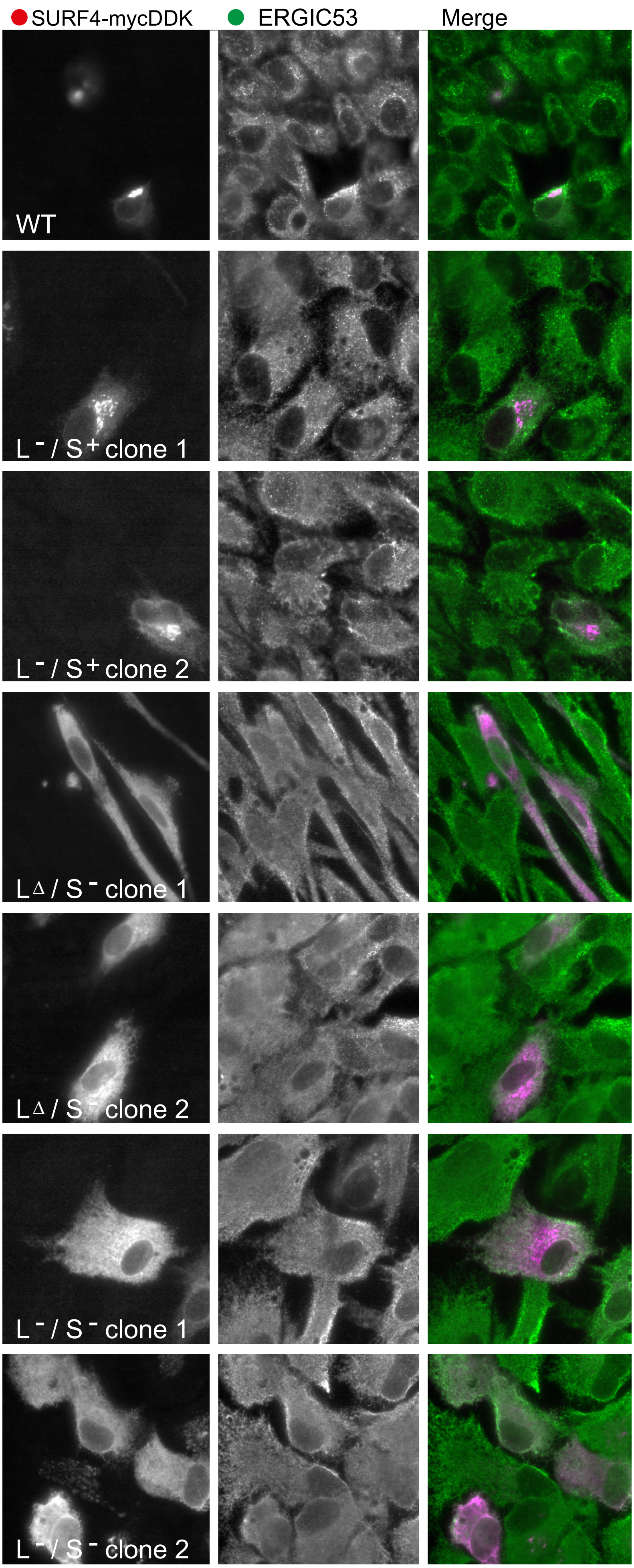

Fig. S4. Localization of SURF4-mycDDK expressed in each cell line. Cells were transfected with SURF4-mycDDK were then fixed and processed for immunofluorescence using to detect transfected cells and endogenous ERGIC53. >20 cells analysed from a single experiment.

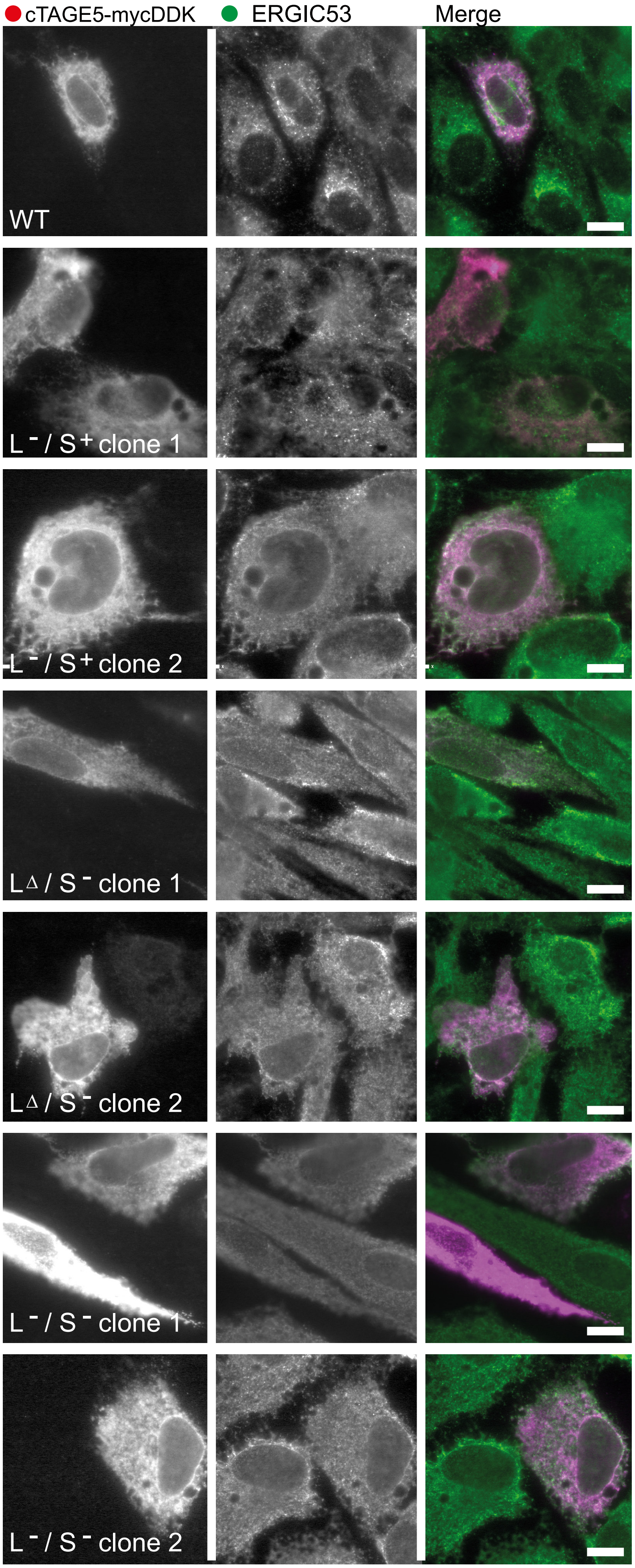

Journal of Cell Science • Supplementary information

Fig. S5. Expression of cTAGE5-mycDDK cannot restore the typical localization of ERGIC53 to TANGO1 knockout cell lines. Transfected cells were detected using the FLAG epitope tag and co-labelled to detect endogenous ERGIC53. >20 cells analysed from a single experiment.
